# Collision activity during training increases total energy expenditure measured via doubly labelled water

**DOI:** 10.1007/s00421-018-3846-7

**Published:** 2018-03-22

**Authors:** Nessan Costello, Kevin Deighton, Thomas Preston, Jamie Matu, Joshua Rowe, Thomas Sawczuk, Matt Halkier, Dale B. Read, Daniel Weaving, Ben Jones

**Affiliations:** 1 Carnegie School of Sport, Institute for Sport, Physical Activity and Leisure, Leeds Beckett University, Leeds, UK; 2 Leeds Rhinos RLFC, Leeds, UK; 3 Stable Isotope Biochemistry Laboratory, Scottish Universities Environmental Research Centre, Rankine Avenue, Scottish Enterprise Technology Park, East Kilbride, Scotland UK; 4 Queen Ethelburga’s School, York, UK; 5 Yorkshire Carnegie RUFC, Leeds, UK; 6 The Rugby Football League, Leeds, UK

**Keywords:** Nutrition, Recovery, Contact, Rugby

## Abstract

**Purpose:**

Collision sports are characterised by frequent high-intensity collisions that induce substantial muscle damage, potentially increasing the energetic cost of recovery. Therefore, this study investigated the energetic cost of collision-based activity for the first time across any sport.

**Methods:**

Using a randomised crossover design, six professional young male rugby league players completed two different 5-day pre-season training microcycles. Players completed either a collision (COLL; 20 competitive one-on-one collisions) or non-collision (nCOLL; matched for kinematic demands, excluding collisions) training session on the first day of each microcycle, exactly 7 days apart. All remaining training sessions were matched and did not involve any collision-based activity. Total energy expenditure was measured using doubly labelled water, the literature gold standard.

**Results:**

Collisions resulted in a very likely higher (4.96 ± 0.97 MJ; ES = 0.30 ± 0.07; *p* = 0.0021) total energy expenditure across the 5-day COLL training microcycle (95.07 ± 16.66 MJ) compared with the nCOLL training microcycle (90.34 ± 16.97 MJ). The COLL training session also resulted in a very likely higher (200 ± 102 AU; ES = 1.43 ± 0.74; *p* = 0.007) session rating of perceived exertion and a very likely greater (− 14.6 ± 3.3%; ES = − 1.60 ± 0.51; *p* = 0.002) decrease in wellbeing 24 h later.

**Conclusions:**

A single collision training session considerably increased total energy expenditure. This may explain the large energy expenditures of collision-sport athletes, which appear to exceed kinematic training and match demands. These findings suggest fuelling professional collision-sport athletes appropriately for the “muscle damage caused” alongside the kinematic “work required”.

**Electronic supplementary material:**

The online version of this article (10.1007/s00421-018-3846-7) contains supplementary material, which is available to authorized users.

## Introduction

Team-based collision sports such as rugby league, rugby union, rugby sevens, American Football and Australian Football are defined by frequent high-intensity collisions (Clarke et al. 2017; Edwards et al. 2017; Gray and Jenkins 2010; Hausler et al. 2016; Quarrie et al. 2013). Collision events include tackling, isometric holding, blocking, wrestling, hit-ups and impacts with the playing surface (Naughton et al. 2017). Both collision frequency and magnitude are sport, match and position specific; however, typically reflect increases in physical fitness, anthropometric quality and playing ability within professional athlete cohorts (Clarke et al. 2017; Hausler et al. 2016). Accordingly, high-intensity collisions peak at nearly three events per minute within professional match play (Hausler et al. 2016), producing impacts that often exceed ‘severe’ gravitational forces (> 10 G; Edwards et al. 2017; Hausler et al. 2016). Unsurprisingly, collision event success has been associated with both increased performance (Tim and Peter 2009) and a decreased injury risk (Tucker et al. 2017), defining training preparation, match performance and subsequent recovery of collision-based sports (Clarke et al. 2017; Edwards et al. 2017; Gray and Jenkins 2010; Hausler et al. 2016; Quarrie et al. 2013).

Collisions induce substantial muscle damage (collision-induced muscle damage; CIMD; Naughton et al. 2017), which may increase the energetic cost of recovery. Both collision frequency and magnitude strongly correlate with the muscle damage response following training and match play (Roe et al. 2017). Repeated, high-impact collisions impair muscle integrity (Tavares et al. 2017), disturbing biochemical (Hoffman et al. 2002) and endocrine homeostasis (McLellan et al. 2011). Subsequently, an acute phase inflammatory response and tissue remodelling period are initiated (Hyldahl and Hubal 2014), substantially upregulating whole body protein turnover (Peake et al. 2017). Such dramatic perturbations of homeostasis are likely to be energetically expensive (Welle and Nair 1990), potentially increasing post-exercise metabolism (Burt et al. 2014) and the energetic cost of recovery for up to 120 h after competitive match play (McLellan et al. 2011), throughout the season (Fletcher et al. 2016).

To safeguard the energy availability of professional collision-sport athletes, it is vital to quantify the energetic costs of collision-based activity. Professional collision-sport athletes have distinct total energy expenditures (TEE) (Morehen et al. 2016), which appear to exceed the energetic demands of similar professional, non-collision sports (i.e. soccer; Anderson et al. 2017). These energetic differences have been observed despite non-collision athletes competing in additional match play across data collection periods (Anderson et al. 2017). This suggests that the unique TEEs of professional collision-sport athletes may exceed the kinematic demands of both training and match play (Morehen et al. 2016), possibly a result of substantial CIMD. Successively, to maximise the health, development and performance of professional collision-sport athletes, investigation into the energetic costs of collisions is required (Mountjoy et al. 2014).

Therefore, this study investigated the energetic cost of collisions for the first time across any sport. Total energy expenditure was measured via doubly labelled water (DLW), the literature gold standard (Westerterp 2017). We hypothesised that the inclusion of 20 competitive collisions would increase TEE across otherwise matched 5-day training microcycles.

## Methods

### Participants

Six healthy, professional young (age range 16–18 years) male RL players [mean ± SD, age; 17.2 ± 0.7 years, height; 178.2 ± 9.4 cm, body mass (BM); 87.3 ± 14.9 kg] completed the study. Eight participants were originally recruited; however, two participants were excluded from analysis because they sustained injuries outside of the COLL or nCOLL training intervention. Participants were chosen from a range of playing positions including Loose Forward, Prop Forward, Half Back, Hooker and Wing. All participants provided written informed consent, prior to volunteering. Ethics approval was granted by the Carnegie Faculty Research Ethics Committee (Leeds Beckett University, UK).

### Design

A randomised crossover design was utilised to assess the magnitude of change in TEE across two different 5-day pre-season training microcycles. Each microcycle included the COLL or nCOLL training intervention, four matched resistance-training sessions, three field sessions and one rest day (Table 1). The COLL and nCOLL training sessions took place on the morning of the first day of both training microcycles, exactly 7 days apart (06:30–07:15). The crossover design was not counterbalanced due to two participant injuries, which resulted in four participants completing the COLL training intervention first and two participants completing the nCOLL training intervention first. The resting metabolic rate (RMR) of participants was assessed 1 day prior to the start of each training microcycle. Internal, external and home-based loads were recorded throughout each assessment microcycle. The study was conducted during the sixth and seventh week of a pre-season period to ensure that participants were adequately conditioned, preventing a possible exaggerated fatigue or energetic response. Pre-existing muscle damage was minimised by avoiding collisions in the week prior to the first training microcycle. Participants abstained from exercise on the day prior to each assessment period.

**Table 1:** Training schedule and data collection protocol across training microcycles

	− 24 h	Intervention	+ 24 h	+ 48 h	+ 72 h	+ 96 h
AM	RMR (06:30–11:00)	Resistance training and intervention (06:30–07:30)	Rest	Rest	Rest	Resistance training and field (06:30–07:30)
	Baseline urine (06:30–11:00)					
	Anthropometric height and weight (06:30–11:00)					
	DLW dose (06:30–11:00)					
	Urine sample (07:30–11:00)					
PM	Rest	Rest	Resistance training and field (16:00–18:30)	Rest	Resistance training and field (16:00–18:30)	Rest
	Wellbeing and urine sample (22:00)	Wellbeing and urine sample (22:00)	Wellbeing and urine sample (22:00)	Wellbeing and urine sample (22:00)	Wellbeing and urine sample (22:00)	Wellbeing and urine sample (22:00)

Training days are shown in relation to the COLL training intervention rather than days of the week. Times in parentheses represent length of the training session or data collection period. The training schedule represents a typical 5-day pre-season training microcycle

### Collision training session intervention

The COLL session was comprised of 20 full contact collisions divided into 10 one-on-one tackles (i.e. tackling an opponent) and 10 one-on-one hit-ups (i.e. being tackled by an opponent). Twenty collisions represent match demands similar to those reported for professional RL (Hausler et al. 2016), rugby union (Quarrie et al. 2013), rugby sevens (Clarke et al. 2017), Australian football (Gray and Jenkins 2010) and American football (Edwards et al. 2017). The corresponding nCOLL session replicated the COLL session exactly, however, without collisions. Participants performed the same drill but accelerated past each other without making contact, thus replicating kinematic demands between groups (supplementary material, Table 2). Prior to either session, participants performed a standardised warm-up overseen by the lead strength and conditioning coach, which included two submaximal shoulder bag tackles on each shoulder to prepare participants for collision.

**Fig. 1:**
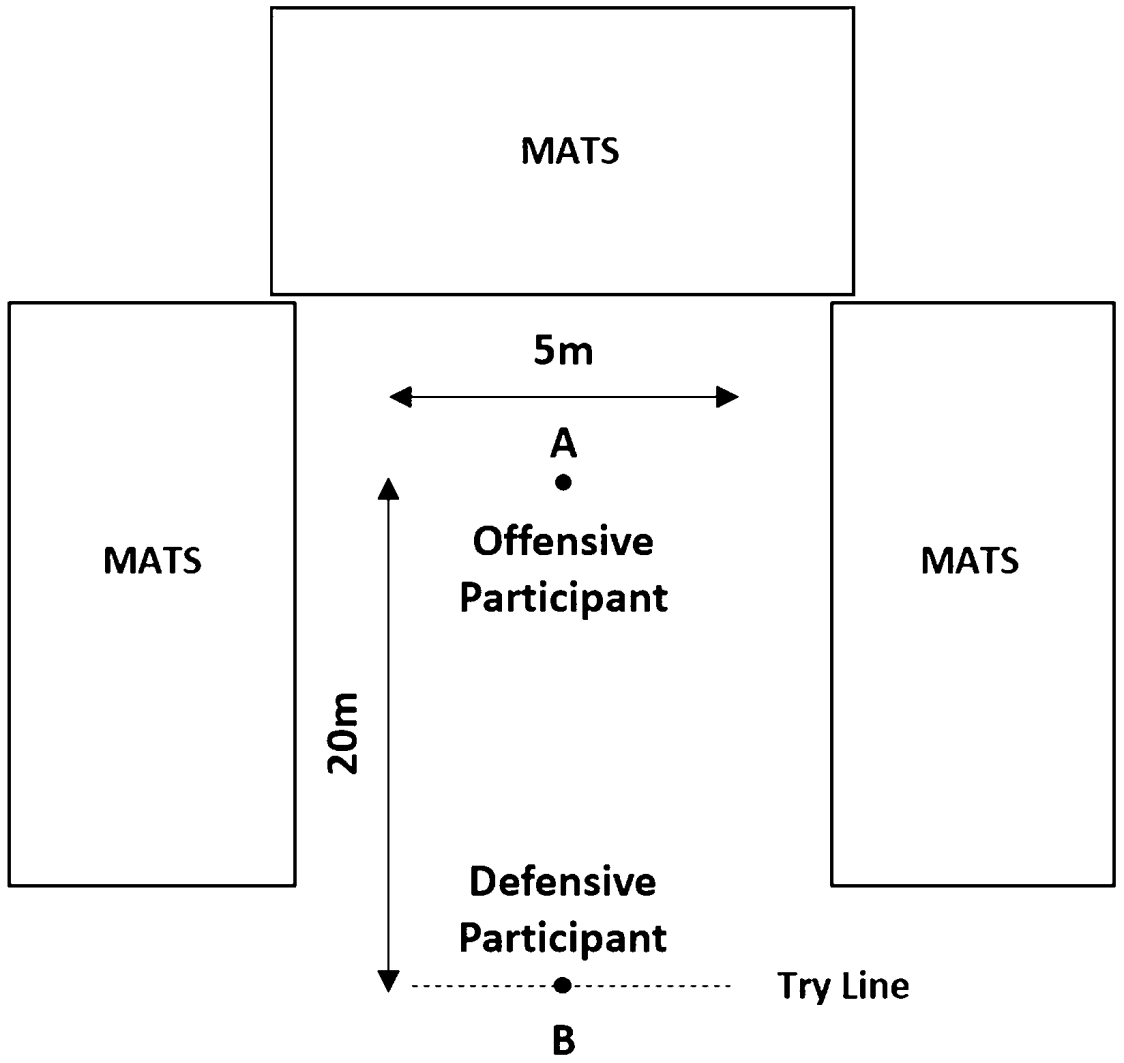
COLL training session intervention

The COLL session replicated a typical collision-based training session (Fig. 1). The drill utilised a 20 m × 5 m grid area (length × width). A grid width of 5 m was chosen so that participants could not avoid collision. The offensive participant started on cone ‘A’ (Fig. 1) with the ball in hand. On the blow of the coach’s whistle, the participant accelerated forward and tried to score over the try line of the opposing defensive participant (dashed line; Fig. 1). The defensive participant started on cone ‘B’, and on the same starting whistle accelerated forward and tried to tackle the offensive participant, driving them back onto the mats on either side or behind the offensive participant. The drill was repeated until all participants had completed 10 tackles and 10 hit-ups, as recorded by the lead researcher. The tackle count included ineffective tackles, as long as participants made contact. The drill was competitive, with participants verbally encouraged to try and beat their opposing participant. Professional coaches directed both sessions to ensure session safety and ecological validity.

### Doubly labelled water

#### Stable isotope doses

Two bolus doses consisting of deuterium (^2^H) and oxygen (^18^O) stable isotopes were prepared for each participant. Doses were calculated relative to the largest BM of any participant included in the study (Schoeller et al. 1980). Specifically, this included ^2^H_2_O (99 atom%) based on 0.14 g kg^−1^ and H_2_^18^O (10 atom%) based on 0.90 g kg^−1^ of BM.

#### DLW administration, urine collections and analyses

Dose administrations were made under close supervision 1 day prior to the start of either trial period, after morning RMR assessment. Participants were weighed wearing shorts only to the nearest 0.1 kg (SECA, Birmingham, UK). A baseline urine sample was provided before oral consumption of a single bolus of DLW (^2^H_2_^18^O). To ensure consumption of the whole bolus, the dose bottles were washed twice with additional water that participants also consumed. Baseline enrichment was determined from a later urine sample provided by participants at 22:00, allowing for total body water (TBW) equilibrium (Schoeller et al. 1980). This protocol was repeated exactly for the second dose 7 days later.

Participants provided daily urine samples at 22:00 across the entire data collection period. Samples were collected directly into two date-, time- and participant ID-registered 5-mL cryovials. Cryovials were then immediately placed in date- and participant ID-labelled ziplock bags and stored in the home fridges of participants. The following morning, participants provided the lead researcher with the vials, which were then filtered in compliance with the Human Tissue Act, frozen at − 40 °C and stored. Analysis of ^2^H and ^18^O abundance was performed following gas exchange (HYDRA 20–22 IRMS, SerCon, Crewe UK). Urine and standards were analysed with two measurements of duplicate samples. All data were imported into a Microsoft Excel template where the calculation of TBW, TEE and quality control parameters could be performed.

#### Total body water and total energy expenditure calculations

Participant TBW and TEE were calculated specifically for each 5-day assessment microcycle. Participant TBW was calculated from stable isotope dilution spaces based on the intercept of the elimination plot of deuterium and TEE was determined from the stable isotope elimination rate constants and “pool space” (IAEA 2009). Specific TEE values were then calculated (Goran et al. 1994). The average pool space ratio was 1.043 and the average tracer elimination rate ratio (kO/kD) was 1.348. Tracer enrichment in body water remained above the minimum recommendation throughout the study (IAEA 2009). The average resampling error on TBW and TEE was 1.4 and 6.8%, respectively. The Pearson product moment correlation of the tracer elimination plots was greater than 0.99 in all cases. A respiratory quotient of 0.85 was assumed.

### Resting metabolic rate

The RMR of participants was assessed 1 day prior to the start of each training microcycle. Participants underwent an overnight fast and 15-min enforced rest period before the beginning of a 15-min assessment. The assessment occurred within a mildly lit and temperate room (21–23 °C) with participants lying quietly in a supine position (Compher et al. 2006). Expired gas was analysed using an online gas analyser (Metalyzer 3BR3, Cortex, Leipzig, Germany). The gas analyser was calibrated as per the manufacturer’s guidelines using two known concentrations of each gas (ambient and 15% O_2_ and ambient and 5% CO_2_), daily barometric pressure and a 3-L volume syringe. Participants wore a facemask connected to a gas analyser for online breath-by-breath analysis. Data were subsequently averaged every 30 s to remove artefacts and exported to Microsoft Excel (2016, Seattle, USA). The respiratory exchange ratio was determined from V̇O_2_ and V̇CO_2_ measurements (Frayn 1983). Energy expenditure was estimated from substrate oxidation rates and expressed per 24 h, using an energy value for carbohydrate and fat of 3.75 and 9 kcal, respectively (Southgate and Durnin 1970).

### Training and home-based loads

Six training and eight home-based load variables were collected throughout each assessment microcycle and are presented in the supplementary material (Tables 2–4). The six training loads included one internal and five external loads and were collected via sessional ratings of perceived exertion (sRPE) and micro-technological units, respectively. The eight home-based loads were collected via SenseWear Armbands (SWA). Collisions during the COLL and nCOLL training sessions were also filmed (video camera; SONY HVR-HD1000) and coded into tackles and hit-ups by an expert analyst using Sportscode (Sportec, NSW). This ensured that each participant performed the required number of collisions.

Internal loads were assessed by sRPE. Participants reported their RPE 15 min after the completion of each training session using a modified Borg scale, in isolation from other participants (Foster et al. 2001). RPE was multiplied by the duration of the training session to calculate the training load in arbitrary units (sRPE; AU) (Foster et al. 2001). Individual training session sRPE were then summated to provide an overall weekly load across COLL and nCOLL microcycles, due to the inability of micro-technological units or SWA to capture the entire weekly training load (Foster et al. 2001).

External training demands were assessed across all training sessions via micro-technological units. Units housed a global positioning system (GPS) and accelerometer (Optimeye S5, Catapult Innovations, Melbourne, Australia) sampling at 10 and 100 Hz, respectively. All units were turned on prior to session warm-ups and turned off immediately following session completion. Data were then downloaded and analysed using Catapult Sprint software [Catapult Innovations, Melbourne, Australia; number of satellites, version 5.1.7, 15 (3); horizontal dilution of precision 0.8 (0.6)]. 10 Hz GPS units have been shown to provide accurate assessment of total distance and high-intensity activity for team sport athletes (Rampinini et al. 2015).

Training loads accumulated away from the club (i.e. home-based loads) were quantified using SWA (SenseWear Professional version 6.1; BodyMedia, Pittsburgh, PA, USA). These were worn at all times by participants except for training sessions and any periods spent submerged in water (i.e. showers, baths). Data were downloaded and analysed using SenseWear computer software (BodyMedia, USA). SenseWear armbands provide valid energetic assessments of low-intensity exercise, such as home-based loads accumulated outside of training sessions (Drenowatz and Eisenmann 2011).

### Wellbeing

A six-item adapted questionnaire (McLean et al. 2010) was used to rate the sleep quality, fatigue, muscle soreness (upper and lower body), stress and mood of participants on a five-point Likert scale. Each item was rated from one to five in one score increment and overall wellbeing was assessed by adding up all six scores. The questionnaire was administered in isolation to prevent peer influence and has been previously used to assess the wellbeing of professional collision-sport athletes (McLean et al. 2010; Fletcher et al. 2016; Roe et al. 2017).

### Statistical analyses

Both null-hypothesis significance testing and magnitude-based inferences (MBI) were used to analyse all trial-based differences. In particular, MBI were included to promote direct interpretation of observed changes and whether observed changes were meaningful (Hopkins et al. 2009). For null-hypothesis significance testing, statistical significance was assumed at 5% (*p* < 0.05). For MBI, the threshold for a change to be considered practically important (the smallest worthwhile change) was set at 0.2 × between subject SD, based on Cohen’s *d* effect size (ES) principle (Hopkins et al. 2009). Thresholds for ES were set as < 0.2 trivial, 0.2–0.59 small, 0.6–1.19 moderate, and 1.2–2.0 large (Hopkins et al. 2009). The probability that the magnitude of difference was greater than the practically important threshold was rated as < 0.5%, almost certainly not; 0.5–4.9%, very unlikely; 5–24.9%, unlikely; 25–74.9%, possibly; 75–94.9%, likely; 95–99.5%, very likely; > 99.5%, almost certainly (Hopkins et al. 2009). Where the 90% CI crossed both the upper and lower boundaries of the practically important threshold (ES ± 0.2), the magnitude of change was described as unclear. Paired *t* test analyses were carried out in IBM SPSS statistics for Windows version 24 (SPSS Inc, Chicago, USA). All MBI calculations were completed using a predesigned spreadsheet (Hopkins 2006).

A linear mixed model was used to analyse differences in TEE in SAS University Edition (SAS Institute Inc., Cary, NC). The linear mixed model incorporated training and home-based loads accumulated outside of the COLL or nCOLL training session intervention as covariates, thus statistically accounting for differences between microcycles. To reduce the number of covariates and multicollinearity between variables, two separate principle component analyses were performed to determine which of the six training and eight home-based loads accounted for the largest variance outside of the COLL or nCOLL training intervention. Analyses identified PlayerLoad 2D and METS_AVG_ as the predominant training and home-based load variables, respectively. Consequently, PlayerLoad 2D and METS_AVG_ were added to the linear mixed model as training and home-based load covariates. The training intervention (COLL or nCOLL) was added as a fixed effect and participant was added as a random effect. Least squared mean differences were used to quantify the difference between training microcycles. Addition of covariates was evaluated as a two SD difference in the mean effect. Covariate-adjusted TEE data are presented in the manuscript, whereas raw TEE data are presented in supplementary material (Table 1). The inclusion of covariates did not alter the interpretation of the findings.

To calculate power, the expected difference in TEE was based on previous findings from a comparable field study utilising DLW in senior professional RL players (Morehen et al. 2016). Based on this and an alpha value of 5%, a sample size of six participants provides > 93% power to detect a difference in TEE between sessions. Consequently, the sample size employed was deemed sufficient to detect a significant difference. All calculations were performed using G*power (Faul et al. 2007). Data are presented as mean ± standard deviation.

## Results

### Energy expenditure

**Fig. 2:**
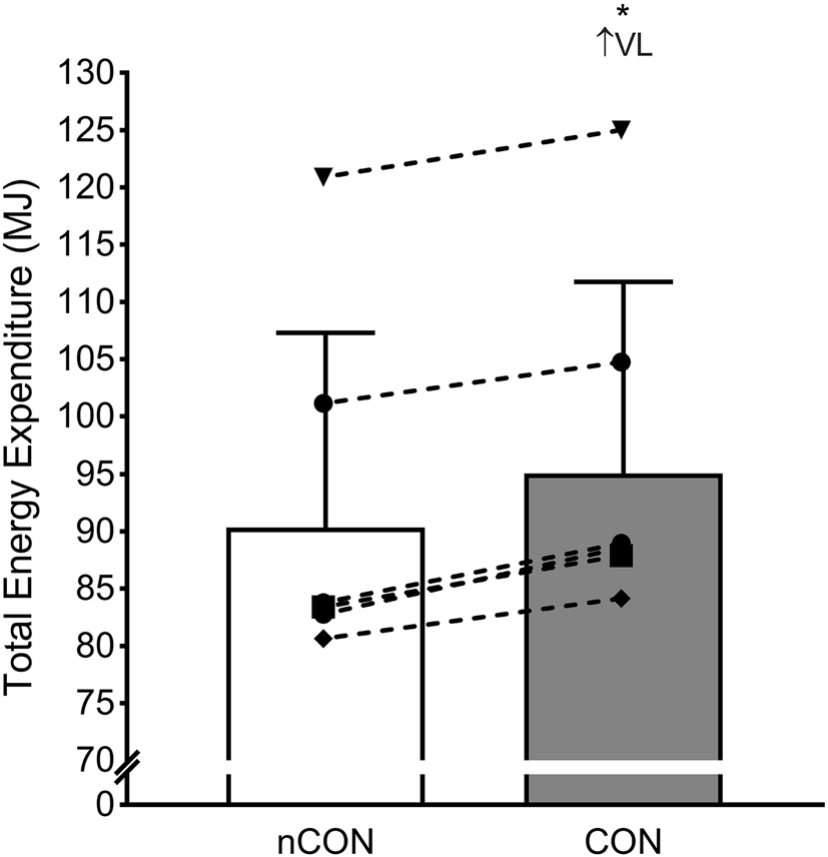
Summated TEE across nCOLL and COLL training microcycles. Bar charts and dashed lines represent mean and individual TEE changes, respectively. Above graph, ratings of probability refer to within-group changes: VL, very likely and ↑, increase. Asterisk indicates a statistically significant difference (*p* < 0.05)

Individual and mean TEE data are presented in Fig. 2. Differences in RMR 1 day prior to the nCOLL training period (11.11 ± 2.16 MJ) were unclear (0.18 ± 0.84 MJ; ES = 0.03 ± 1.08; *p* = 0.622) compared with the COLL training period (11.29 ± 2.25 MJ). There was a very likely higher (4.96 ± 0.97 MJ; ES = 0.30 ± 0.07; *p* = 0.0021) TEE across the 5-day training period including the COLL training session (95.07 ± 16.66 MJ) compared with the nCOLL training session (90.34 ± 16.97 MJ). Differences in total distance during the COLL intervention (1069 ± 61 m) were unclear (47 ± 159 m; ES = − 0.50 ± 1.55; *p* = 0.315; supplementary material, Table 2) compared with the nCOLL intervention (1022 ± 95 m). Differences in total distance accumulated across the 5-day COLL microcycle (9513 ± 640 m) were unclear (305 ± 573 m; ES = 0.39 ± 0.72; *p* = 0.105; supplementary material, Table 3) compared with the 5-day nCOLL microcycle (9818 ± 439 m).

### sRPE and wellbeing

**Fig. 3:**
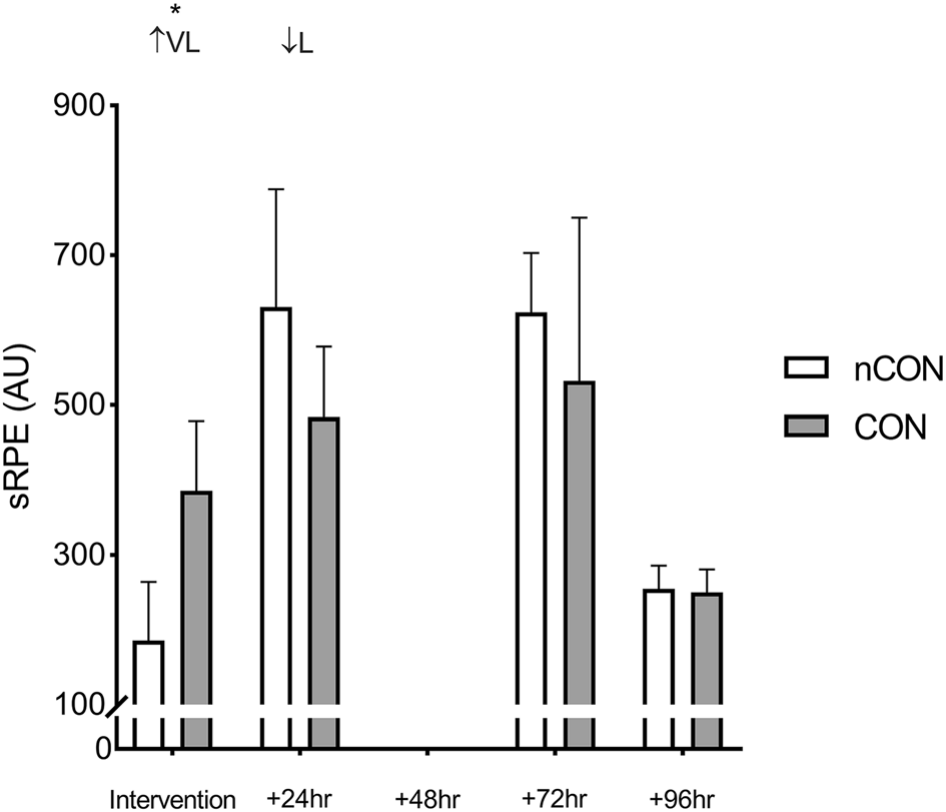
Mean and standard deviation sRPE for individual training days across nCOLL and COLL training microcycles. Above graph, ratings of probability refer to within-group changes: L, likely; VL, very likely; ↑, increase and ↓, decrease. Asterisk indicates a statistically significant difference (*p* < 0.05)

**Fig. 4:**
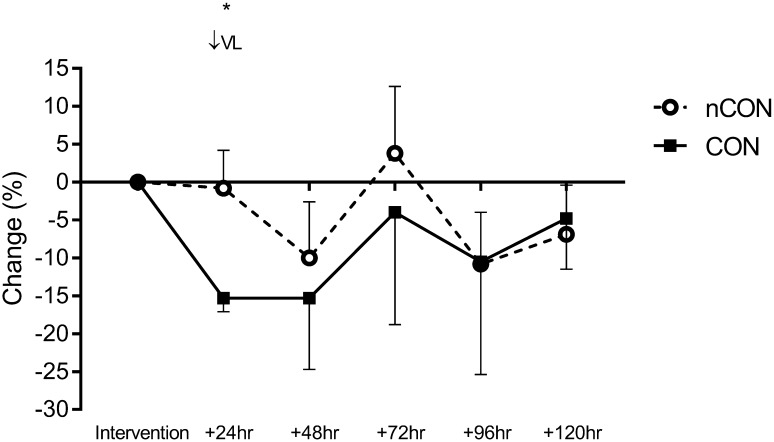
Mean changes in subjective wellbeing across nCOLL and COLL training microcycles. Change data are percentage change with 90% confidence interval bars. Above graph, ratings of probability refer to within-group changes: VL, very likely and ↓, decrease. Asterisk indicates a statistically significant difference *p* < 0.05)

Participant sRPE and wellbeing data are presented in Figs. 3 and 4, respectively. There was a very likely higher (200 ± 102 AU; ES = 1.43 ± 0.74; *p* = 0.007) sRPE during the COLL training session and a very likely greater (− 14.6 ± 3.3%; ES = − 1.60 ± 0.51; *p* = 0.002) decrease in wellbeing 24 h after the COLL training session compared with the nCOLL training session. Differences in accumulated sRPE across the COLL training microcycle (1785 ± 236 AU) were unclear (89 ± 327 AU; ES = 0.30 ± 0.84; *p* = 0.533; supplementary material, Table 3) compared with the nCOLL training microcycle (1696 ± 253 AU).

## Discussion

This is the first study to investigate the energetic cost of collisions across any sport. The findings demonstrate that twenty competitive one-on-one collisions resulted in a very likely higher TEE across otherwise matched 5-day training microcycles. The COLL training session also resulted in a very likely higher sRPE and very likely greater decrease in wellbeing 24 h after session completion. This study provides novel evidence that collisions increase DLW-assessed TEE and may explain the large TEE of collision-sport athletes, which appear to exceed kinematic demands of training and match play. Practically, these findings have immediate implications for coaches, practitioners and athletes operating within collision-based sports.

Twenty competitive one-on-one collisions resulted in a very likely higher TEE, representing a meaningful 5% increase across the 5-day training microcycle. Practically, it is important to consider that professional collision athletes typically experience two collision sessions each week (i.e. two training sessions or one training session plus a match) across pre- and in-season periods (Roe et al. 2017). Therefore, the increased energetic cost of collisions evidenced in this study are likely modest, compared to what collision athletes are actually exposed to across the season. Accordingly, coaches and practitioners should ensure sufficient energy intake following challenging collision-based activity to safeguard the energy availability of professional collision athletes (Mountjoy et al. 2014) across the season (Fletcher et al. 2016).

This study provides novel evidence that the increased energetic costs of collisions may be responsible for the distinct TEEs of professional collision-sport athletes (Morehen et al. 2016), which appear to exceed the kinematic demands of training or match play (Anderson et al. 2017). Despite competing in one less competitive match across the data collection period, professional senior collision athletes (e.g. rugby players) report substantially higher DLW assessed TEEs (5780 kcal day^−1^; Morehen et al. 2016) than professional senior non-collision field-based athletes (e.g. soccer players; 3566 kcal day^−1^; Anderson et al. 2017). This conclusion is strengthened in this study by matching training sessions across microcycles and statistically controlling for training and home-based loads as covariates within the analyses. The results challenge previous research suggestions that potential increases in non-exercise activity thermogenesis or internal training loads (sRPE) could be responsible for the unique TEEs of collision-sport athletes (Morehen et al. 2016).

We propose that recovery from CIMD drives observed increases in TEE, rather than collision-based kinematic demands. Morehen et al. (2016) quantified the TEE of professional senior collision-sport athletes across a 2-week period, observing a very likely 35.3% increase in TEE from week 1 to week 2. Participants competed in two competitive matches across the data collection period, on the final day of both weeks (Sunday). Consequently, TEE measured within week 1 would have included kinematic collision demands from the match on day 7 and recovery costs accrued until 12:00 p.m on Sunday night. In contrast, the very likely higher TEE measured within week 2 would have included kinematic collision demands from the match on day 14, on top of nearly all of the hypothesised recovery costs from the match in week 1. Accordingly, it seems likely that the observed 35.3% increase in TEE during week 2 (Morehen et al. 2016) represents the increased energetic cost of recovery from CIMD sustained during the match in week 1. Interestingly, the observed increase in TEE is considerably larger than the 5% observed in this study. This could possibly represent the higher metabolic cost of recovery from CIMD sustained during competitive match play within a senior population, compared to training-based activity within an adolescent population.

The increased energetic cost of collisions observed in this study could be caused by increases in whole body protein turnover in response to CIMD (Damas et al. 2016), suggesting that professional collision-sport athletes should fuel appropriately for the “muscle damage caused” alongside the kinematic “work required” (Impey et al. 2016). The COLL training session resulted in a very likely greater reduction in self-perceived wellbeing compared to the nCOLL training session, suggesting that substantial CIMD occurred (McLean et al. 2010; Fletcher et al. 2016; Roe et al. 2017). Muscle damage disturbs homeostasis initiating an inflammatory response (Hyldahl and Hubal 2014), which significantly increases whole body protein turnover (Peake et al. 2017) above that observed via muscular hypertrophy alone (Damas et al. 2016). Protein metabolism is an energetically expensive process (Welle and Nair 1990), elevating RMR (Welle and Nair 1990) for up to 48 h after muscle damaging exercise (Burt et al. 2014). Interestingly, muscle damage follows a similar trajectory for up to 120 h after strenuous collision-based activity (McLellan et al. 2011), possibly highlighting a mirrored energetic response. Such a relationship is commonly reported in the literature (McLellan et al. 2011; Naughton et al. 2017; Roe et al. 2017; Tavares et al. 2017) and could provide practitioners with a practical day-by-day surrogate measure of increased energetic demands in response to CIMD (McLean et al. 2010).

Future research should seek to progress these initial findings by establishing the causal mechanism for the observed increase in TEE. Likewise, determining the sensitivity of standardised changes in self-perceived wellbeing in relation to increased energetic demands, alongside specific macronutrient requirements in response to CIMD (e.g. protein), would be of great benefit to practitioners. Such research would likely benefit from a larger sample size, despite six participants providing sufficient power to detect significant differences in TEE in this study. Moreover, combined utilisation of objective and subjective markers of muscle damage would increase confidence in outcome measures and overall study conclusions.

In conclusion, this study provides novel insights into the energetic costs of collisions for the first time across any sport. Study findings demonstrate that a single COLL training session resulted in a very likely higher TEE across an otherwise matched 5-day training microcycle. The utilisation of gold standard assessment techniques and inclusion of training and home-based load covariates within a mixed model analysis represents unique control of extraneous variables within an ecologically valid research protocol. These findings elucidate the distinct TEE of professional collision athletes for the first time, which appears to exceed the kinematic demands of training or match play. Accordingly, coaches and practitioners should ensure appropriate energy intake following challenging collision-based activity to safeguard the energy availability of professional collision athletes across the season.

### Availability of data and materials

Results are presented clearly, honestly, and without fabrication, falsification, or inappropriate data manipulation.

## Electronic supplementary material

Below is the link to the electronic supplementary material.

Supplementary material 1 (DOCX 31 KB)

## Abbreviations

^18^O: Oxygen
^2^H: Deuterium
BM: Body mass
COLL: Collision training session
CIMD: Collision-induced muscle damage
DLW: Doubly labelled water
ES: Effect size
FFM: Fat-free mass
FM: Fat mass
GPS: Global positioning system
MBI: Magnitude-based inferences
METSAVG: Average metabolic equivalents
nCOLL: Non-collision training session
RL: Rugby league
RMR: Resting metabolic rate
sRPE: Sessional ratings of perceived exertion
SWA: SenseWear Armbands
TBW: Total body water
TEE: Total energy expenditure
