# Supplementary material for: Collision activity during training increases total energy expenditure measured via doubly labelled water
Source: Eur J Appl Physiol. 2018 Mar 22;118(6):1169–77. doi: 10.1007/s00421-018-3846-7 (PMC5966477; doi:10.1007/s00421-018-3846-7)
Supplement: Supplementary file 1 — Supplementary material 1 (DOCX 31 KB) [file 421_2018_3846_MOESM1_ESM.docx]

**Article title**: Collisions increase total energy expenditure measured via doubly labelled water.

**Journal:** European Journal of Applied Physiology

**Authors names**: Nessan Costello, Kevin Deighton, Thomas Preston, Jamie Matu, Joshua Rowe, Thomas Sawczuk, Matt Halkier, Dale B. Read, Daniel Weaving, Ben Jones.

**Corresponding author**: Nessan Costello, Institute for Sport Physical Activity & Leisure, Leeds Beckett University, Leeds, LS6 3QS, United Kingdom (email: [N.Costello@leedsbeckett.ac.uk](mailto:N.Costello@leedsbeckett.ac.uk)).

| **Total Energy Expenditure** | **Collision Training Session** | **Non-Collision Training Session** | **Significance (*p*) Value** |
| --- | --- | --- | --- |
| MJ | 19.01 ± 3.33 | 18.07 ± 3.40 | < 0.001 |
| kcal | 4541 ± 796 | 4315 ± 811 |  |

**Supplementary Table 1.** Raw total energy expenditure across five-day collision (COLL) and non-collision (nCOLL) training microcycles.

| **Internal & External Training Loads** | **Collision Training Session** | **Non-Collision Training Session** | **Significance (*p*) Values** |
| --- | --- | --- | --- |
| sRPE (AU) | 386 ± 93 | 186 ± 78 | = 0.007 |
| Total Distance (m) | 1069 ± 61 | 1022 ± 95 | = 0.315 |
| High Speed Running (m) | 43 ± 58 | 116 ± 31 | = 0.014 |
| PlayerLoad™ (AU) | 39 ± 60 | 84 ± 6 | = 0.079 |
| PlayerLoad™ 2D (AU) | 93 ± 14 | 51 ± 4 | = 0.001 |
| PlayerLoad™ slow (AU) | 97 ± 20 | 50 ± 4 | = 0.002 |

**Supplementary Table 2.** Internal and external training loads accumulated across the collision (COLL) and non-collision (nCOLL) training session intervention.

| **Internal & External Training Loads** | **Five-Day Collision Microcycle** | **Five-Day Non-Collision Microcycle** | **Significance (*p*) Values** |
| --- | --- | --- | --- |
| sRPE (AU) | 1785 ± 236 | 1696 ± 253 | = 0.533 |
| Total Distance (m) | 9513 ± 640 | 9818 ± 439 | = 0.105 |
| High Speed Running (m) | 594 ± 131 | 594 ± 146 | = 0.993 |
| PlayerLoad™ (AU) | 1056 ± 43 | 1115 ± 109 | = 0.153 |
| PlayerLoad™ 2D (AU) | 635 ± 131 | 649 ± 149 | = 0.615 |
| PlayerLoad™ slow (AU) | 501 ± 65 | 509 ± 69 | = 0.326 |

**Supplementary Table 3.** Internal and external training loads accumulated across five-day collision (COLL) and non-collision (nCOLL) training microcycles, excluding the collision or non-collision training session intervention.

**Supplementary Table 4.** Internal and external home-based loads accumulated across collision (COLL) and non-collision (nCOLL) training microcycles, excluding training sessions.

| **Home-Based Loads** | **Five-Day Collision Microcycle** | **Five-Day Non-Collision Microcycle** | **Significance (*p*) Values** |
| --- | --- | --- | --- |
| Average Physical Activity Level (PAL) | 9.9 ± 0.8 | 10.1 ± 1.0 | = 0.136 |
| Average Metabolic Equivalents (METS_AVG_) | 10.6 ± 1.2 | 10.7 ± 1.5 | = 0.329 |
| Number of Steps | 63254 ± 14077 | 69785 ± 13763 | = 0.113 |
| Sedentary Activity (up to 1.5 METS) | 6241 ± 817 | 7083 ± 885 | = 0.135 |
| Light Activity (1.5-3.0 METS) | 1330 ± 469 | 1339 ± 396 | = 0.936 |
| Moderate Activity (3.0-6.0 METS) | 1074 ± 170 | 1107 ± 148 | = 0.347 |
| Vigorous activity (6.0-9.0 METS) | 74 ± 59 | 99 ± 58 | = 0.040 |
| Very vigorous activity (9.0 METS and higher) | 4.5 ± 10.1 | 4.8 ± 8.5 | = 0.849 |
